# Effects of exercise on vascular remodelling and fetal growth in uncomplicated and abortion-prone mouse pregnancies

**DOI:** 10.1038/s41598-024-83329-z

**Published:** 2024-12-30

**Authors:** Evangeline A. K. Lovell, Shanna L. Hosking, Holly M. Groome, Lachlan M. Moldenhauer, Sarah A. Robertson, Kathryn L. Gatford, Alison S. Care

**Affiliations:** Robinson Research Institute, School of Biomedicine, University of Adelaide, Adelaide, SA Australia

**Keywords:** Immunology, Medical research, Physiology, Reproductive biology

## Abstract

Studies in humans and rodents show exercise in pregnancy can modulate maternal blood pressure, vascular volume, and placental efficiency, but whether exercise affects early uteroplacental vascular adaptations is unknown. To investigate this, CBA/J female mice mated with BALB/c males to generate healthy uncomplicated pregnancies (BALB/c-mated) or mated with DBA/2J males to generate abortion-prone pregnancies (DBA/2J-mated), were subjected to treadmill exercise (5 days/week, 10 m/min, 30 min/day for 6 weeks before and throughout pregnancy), or remained sedentary. In uncomplicated pregnancies, exercise caused symmetric fetal growth restriction in fetuses evidenced by reductions in fetal weight, crown-to-rump length, abdominal girth and biparietal diameter. Placental insufficiency was indicated by reduced fetal: placental weight ratio and increased glycogen cell content in the junctional zone of placentas of exercised BALB/c-mated mice on gestational day (GD)18.5. In abortion-prone pregnancy, exercise increased placental efficiency, but the number of late-pregnancy resorptions were elevated. Effects of paternal genotype independent of exercise were evidenced by a greater number of resorptions, poorer spiral artery remodelling, and larger placentas in the DBA/2J-mated compared to BALB/c-mated mice. Effects of exercise independent of paternal genotype included increased implantation sites at both mid and late pregnancy, accompanied by decreased junctional zone areas of placentas. Our findings show that exercise before and during pregnancy in mice can have different effects on fetal outcomes, depending on the paternal and/or fetal genotype. This suggests that the underlying mechanisms are responsive to fetal cues.

## Introduction

The maternal cardiovascular system undergoes major systemic and local adaptations during pregnancy to enhance the blood supply to the uterus and facilitate fetal nutrient supply via the placenta^1–3^. In women, systemic cardiovascular adaptations to pregnancy include a 30–40% increase in cardiac output and 50% increase in blood volume, as well as increases in heart rate and vasodilation, accompanied by decreased mean arterial pressure and vascular resistance^3,4^. As well as these systemic changes, the vasculature of the uterus undergoes substantial remodelling. In particular, the uterine spiral arteries – which are the terminal branch of the main uterine arteries that extend into the decidual compartment subjacent to the placenta – must adapt to establish adequate blood supply to the placenta and accommodate increasing fetal demand as pregnancy progresses. This remodelling is critical to adequately perfuse the placental intervillous space and facilitate exchange of nutrients and oxygen between the maternal and fetal circulations^1,2^.

Uterine spiral artery remodelling begins in early placental development, when migrating extravillous trophoblast cells invade both the decidual interstitium and the endovascular space within spiral arteries to remodel them from tightly coiled vessels to low resistance and high-capacity vessels with a 4-fold larger capacity^1,5,6^. Spiral artery adaptation to pregnancy involves the actions of uterine natural killer (uNK) cells, abundant immune cells in the uterus that regulate trophoblast cell invasion through effects on vascular endothelial cell activation state^7,8^. Pregnancy complications including preeclampsia and fetal growth restriction are often associated with impaired uterine artery function and inadequate spiral artery remodelling leading to placental insufficiency^9^.

Exercise may be a useful intervention to help prevent or reduce the severity of pregnancy complications such as preeclampsia and fetal growth restriction^10–12^. National guidelines in countries including the UK, USA and Australia recommend that women with uncomplicated pregnancies undertake moderate intensity exercise for 30–45 min/day, up to 5 days/week, involving cycling or other activities that aim to improve muscular and aerobic fitness and flexibility^13–16^. Exercise before and/or during pregnancy in women can improve cardiovascular function and reduce the risks of gestational hypertension^17–19^, gestational diabetes^17,18,20^, postnatal depression^21^, miscarriage^22^, adverse delivery outcomes^23^, preeclampsia^24,25^ and compromised fetal health^19,20,26^. Although guidelines for human pregnancy recommend moderate exercise, randomly controlled trials and case-control studies show that vigorous exercise before and throughout pregnancy also reduces the risk of excessive weight gain, prematurity, and low birth weight^26,27^. Vigorous exercise is therefore not contraindicated for human pregnancy, but rapid changes in activity levels are not recommended without discussion with obstetric care providers^26^. The better pregnancy outcomes seen with exercise are likely to reflect vascular responses to exercise that in turn facilitate placental development and function. Regular exercise during uncomplicated pregnancies in women increases placental vascular volume, placental efficiency, and endothelium-dependent vasodilation in the brachial artery^28,29^. However, the mechanisms by which this occurs – and particularly, whether the same effects on uterine vascular parameters and placental function occur in complicated human pregnancies – have not been reported.

Mice provide a useful animal model to evaluate how exercise benefits pregnancy outcomes, and previous studies point to an interaction between genetic and environmental determinants of reproductive success that interact with exercise to modulate its impact. In mouse models of pregnancy with features of preeclampsia, voluntary exercise before and during pregnancy attenuated or prevented maternal hypertension, proteinuria and cardiac hypertrophy, and improved placental efficiency and fetal growth^30–32^. In obese mice, exercise before and during pregnancy improved placental function by increased vascularisation, in turn preventing fetal overgrowth and improving offspring cardiac health^33,34^. The effects of exercise may be less evident in mice with normal (uncomplicated) pregnancy, where controlled maternal wheel-running exercise did not alter pregnancy rate, litter size or litter survival^35^, and treadmill running exercise also did not change mean arterial pressure or fetal and placental outcomes^30^ despite decreasing placental necrosis^32^ and reducing blood glucose levels^33^. Collectively, these considerations led us to hypothesise that exercise would improve uterine vascular adaptations and therefore pregnancy outcomes, and these effects would be greater in compromised pregnancies.

In this study, we tested this hypothesis in mice by investigating the impact of exercise before and during pregnancy on uterine vascular parameters, and placental and fetal growth, using two different male partner and semi-allogeneic mating combinations that give rise to fetal genotypes associated with uncomplicated pregnancy (CBA/J females mated with BALB/c males) and abortion-prone pregnancy (CBA/J females mated with DBA/2J males). The CBA/J x DBA/2J mating combination is extensively utilised in reproductive biology research to model aspects of abortion-prone pregnancy, with CBA/J x BALB/c matings considered the most appropriate ‘normal’ comparison^36^. BALB/c and DBA/2J males have the same MHC haplotype on different genetic backgrounds, so both mating combinations generate allogeneic pregnancies with a theoretically similar capacity to elicit a maternal immune response. Nevertheless, there are well-described differences in pregnancy outcomes that are linked to an inflammatory phenotype in DBA/2J-mated dams. Both paternal and fetal-placental genotypes contribute to the underlying mechanisms for poor pregnancy outcomes. Induction of immune tolerance at conception is impaired due to altered seminal fluid composition in DBA/2J compared to BALB/c males^37^, and this immune intolerance is subsequently exacerbated by excess placental anaphylatoxin C5a expression, which dysregulates angiogenic factor synthesis^38,39^. As a result, DBA/2J-mated dams exhibit an immune imbalance with elevated Th1 effector T cells, increased fetal resorption, impaired decidual spiral artery remodelling, smaller placentas, and fetal growth restriction, compared to dams mated with BALB/c sires^36,40^. Our results reveal the significance of paternal and/or fetal determinants for modifying the effects of exercise on pregnancy outcomes. We also identify differences in the sensitivity of the uterine vasculature to exercise-induced changes may be a key underlying mechanism.

## Results

Uncomplicated pregnancies were generated by mating CBA/J females with BALB/c males, and abortion-prone pregnancies were generated by mating CBA/J females with DBA/2J males^36^. Mice underwent treadmill exercise (5 days/week, 10 m/min, 30 min/day) for 6 weeks before and throughout pregnancy or remained sedentary. Two cohorts of mice were evaluated – the first at gestational day (GD)9.5–10.5 to measure effects of exercise on uterine artery hemodynamics, maternal body morphometry, pregnancy parameters, and uterine vascular parameters in mid-pregnancy, and the second at GD17-18.5 to measure effects of exercise on maternal body morphometry, pregnancy parameters, fetal and placental outcomes, and uterine artery and umbilical artery hemodynamics in late pregnancy.

### Maternal body morphometry

Gastrocnemius, quadricep, bicep and tricep muscle masses and retroperitoneal fat masses of dams at GD10.5 (Table 1) were unaffected by exercise (*P* = 0.781, *P* = 0.312, *P* = 0.614, *P* = 0.903 and *P* = 0.517 respectively) or male partner genotype (*P* = 0.855, *P* = 0.425, *P* = 0.151, *P* = 0.652, and *P* = 0.806 respectively). Furthermore, the relative muscle mass, fat mass and the fat to muscle ratio of dams at GD10.5 (Table 1) was unaffected by exercise (*P* = 0.270, *P* = 0.594 and *P* = 0.424 respectively) or male genotype (*P* = 0.677, *P* = 0.934 and *P* = 0.899 respectively.

**Table 1:** Relative muscle and fat weights in CBA/J female mice at GD10.5 and GD18.5.

Gestational day	Tissue	Uncomplicated pregnancy (CBA/J x BALB/c)		Abortion-prone pregnancy (CBA/J x DBA/2J)		Significance		
GD10.5	N =	Sedentary (20)	Exercised (5)	Sedentary (9)	Exercised (7)	P_genotype_	P_exercise_	P_interaction_
	Gastrocnemius (g)	0.057 ± 0.009	0.055 ± 0.009	0.055 ± 0.009	0.056 ± 0.008	0.855	0.781	0.737
	Quadricep (g)	0.104 ± 0.013	0.116 ± 0.016	0.106 ± 0.015	0.106 ± 0.016	0.425	0.312	0.276
	Bicep (g)	0.012 ± 0.005	0.012 ± 0.002	0.013 ± 0.003	0.013 ± 0.003	0.151	0.614	0.762
	Tricep (g)	0.070 ± 0.009	0.071 ± 0.009	0.070 ± 0.009	0.068 ± 0.008	0.652	0.903	0.595
	Muscle Mass (%)	2.080 ± 0.192	2.223 ± 0.192	2.172 ± 0.186	2.187 ± 0.193	0.677	0.270	0.341
	Retroperitoneal fat (g)	0.033 ± 0.013	0.031 ± 0.013	0.033 ± 0.012	0.029 ± 0.013	0.806	0.517	0.860
	Retroperitoneal fat Mass (%)	0.138 ± 0.049	0.134 ± 0.049	0.142 ± 0.048	0.127 ± 0.050	0.934	0.594	0.745
	Fat: Muscle Ratio	0.067 ± 0.027	0.060 ± 0.025	0.066 ± 0.024	0.058 ± 0.024	0.899	0.424	0.911
GD18.5	N =	Sedentary (20)	Exercised (6)	Sedentary (7)	Exercised (8)	P_genotype_	P_exercise_	P_interaction_
	Gastrocnemius (g)	0.058 ± 0.009	0.050 ± 0.010^%^	0.049 ± 0.011	0.057 ± 0.011^%^	0.798	0.958	0.024
	Quadricep (g)	0.105 ± 0.009	0.097 ± 0.010^%^	0.099 ± 0.011*	0.112 ± 0.011*^%^	0.238	0.519	0.009
	Bicep (g)	0.011 ± 0.005	0.010 ± 0.002	0.012 ± 0.003	0.014 ± 0.003	0.035	0.736	0.333
	Tricep (g)	0.071 ± 0.009*^%^	0.064 ± 0.007*	0.065 ± 0.008^%^	0.072 ± 0.009	0.825	0.916	0.009
	Muscle Mass (%)	1.708 ± 0.237	1.538 ± 0.230	1.732 ± 0.230	1.731 ± 0.247	0.192	0.302	0.307
	Retroperitoneal fat (g)	0.033 ± 0.013	0.019 ± 0.012	0.036 ± 0.011	0.026 ± 0.011	0.221	0.005	0.579
	Fat Mass (%)	0.113 ± 0.036	0.067 ± 0.037	0.138 ± 0.034	0.089 ± 0.037	0.075	< 0.001	0.941
	Fat: Muscle Ratio	0.068 ± 0.022	0.042 ± 0.025	0.079 ± 0.024	0.051 ± 0.025	0.218	0.002	0.942

Females were mated with BALB/c or DBA/2J males and underwent treadmill exercise or remained sedentary for 6 weeks before and during pregnancy. Weights of gastrocnemius, quadricep, bicep and tricep muscles and retroperitoneal fats were averaged from left and right sides. Muscle mass was calculated as a percentage of the sum of wet weights of left and right gastrocnemius, quadricep, bicep, and tricep muscles of total bodyweight. Fat mass was calculated as a percentage of average retroperitoneal fat of total bodyweight. Data was analysed by linear mixed model including litter size as a covariate and represented as mean ± SD. N in parentheses. Where interactions were present, we performed sub-analyses to assess effects of exercise within each genotype, and effects of genotype within sedentary and exercised groups. *Indicates differences (*P* < 0.05) between sedentary and exercised groups within a genotype; % indicates differences (*P* < 0.05) between genotypes within sedentary or exercised groups.

Effects of exercise on gastrocnemius, quadricep and tricep muscle masses of dams at GD18.5 (Table 1) differed according to male partner genotype (interaction: *P* = 0.024, *P* = 0.009 and *P* = 0.009 respectively). In exercised groups, gastrocnemius and quadricep muscle masses were 12–17% heavier in DBA/2J-mated dams compared to BALB/c-mated dams (*P* = 0.028 and *P* = 0.004 respectively), but within sedentary groups, gastrocnemius and quadricep muscle masses were unchanged by male genotype (*P* = 0.085 and *P* = 0.385 respectively). Within DBA/2J-mated dams, exercise increased quadricep muscle mass by 14% compared to sedentary dams (*P* = 0.034), but exercise was unchanged within BALB/c-mated dams (*P* = 0.194). Furthermore, within DBA/2J-mated dams and BALB/c-mated dams there was no effect of exercise on gastrocnemius muscle (*P* = 0.113 and *P* = 0.096 respectively). Within BALB/c-mated dams, tricep muscles were 11% smaller with exercise (*P* = 0.024) and within DBA/2J-mated dams tricep muscles were unchanged by exercise (*P* = 0.117). Within sedentary groups, tricep muscles were 9% smaller in DBA/2J-mated dams when compared to BALB/c-mated dams (*P* = 0.046), but within exercised groups tricep muscle mass was unaffected by male genotype (*P* = 0.060). Bicep weights were larger in DBA/2J-mated dams compared to BALB/c-mated dams (*P* = 0.035) and unaffected by exercise (*P* = 0.736). The absolute retroperitoneal fat pad mass (Table 1) was lower in exercised than sedentary dams overall (*P* = 0.005) and did not differ according to male partner (*P* = 0.221). The relative fat mass and fat to muscle ratio (Table 1) were 36% smaller in exercised compared to sedentary dams overall (*P* < 0.001 and *P* = 0.002 respectively) but did not differ according to male partner (*P* = 0.075 and *P* = 0.218 respectively). Furthermore, the relative muscle mass at GD18.5 (Table 1) was unaffected by exercise (*P* = 0.302) or male partner genotype (*P* = 0.192.

### Mid-gestation pregnancy outcomes

**Fig. 1:**
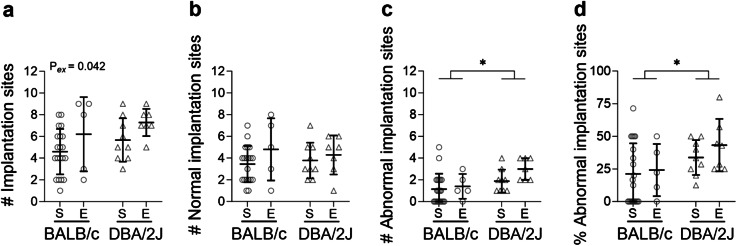
Impact of exercise and paternal genotype on pregnancy outcomes at mid-pregnancy. The total number of implantation sites (**A**), number of normal implantation sites (**B**), number of abnormal implantation sites (**C**) and the proportion of abnormal implantation sites (**D**) per dam for sedentary (S) or exercised (E) CBA/J x BALB/c mice (circles) or abortion-prone CBA/J x DBA/2J mice (triangles) in mid-pregnancy (*N* = 5–20 dams/group). Data was analysed by linear mixed model. Symbols show outcomes from each dam and bars and whiskers indicate mean ± SD within each group. Differences between male genotypes are indicated by *P < 0.05; annotations (P_ex_) show significant effects of exercise.

Exercise did not affect GD10.5 pregnancy rates measured as a proportion of plugged BALB/c-mated dams (sedentary: 22/29 = 76%; exercised: 7/13 = 54%; *P* = 0.154). All plugged DBA/2J-mated dams were pregnant when assessed at GD10.5 (9 sedentary, 7 exercised). The total number of implantation sites (Fig. 1A) at GD10.5 was 49% higher in exercised than sedentary dams overall (*P* = 0.042) and did not differ according to male partner partner genotype (*P* = 0.165). The number of normal implantation sites per dam (Fig. 1B) was unaffected by exercise (*P* = 0.166) or male genotype (*P* = 0.888). The number (Fig. 1C) and proportion (Fig. 1D) of abnormal implantation sites were higher in DBA/2J-mated than BALB/c-mated dams (*P* = 0.013 and *P* = 0.038 respectively) and unaffected by exercise (*P* = 0.136 and *P* = 0.400 respectively).

### Mid-pregnancy vascular parameters

**Fig. 2:**
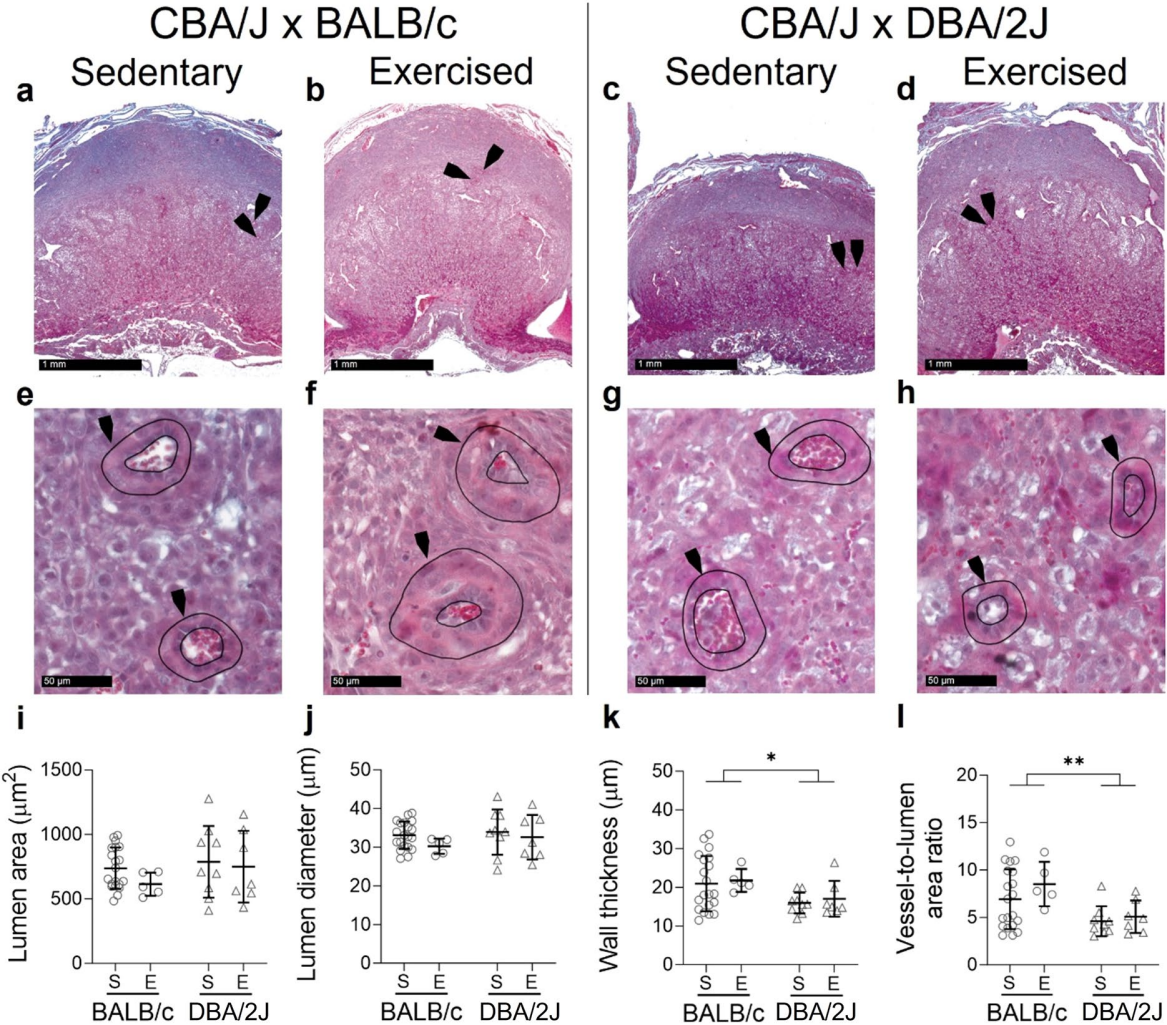
Impact of exercise and paternal genotype on decidual spiral artery remodelling at mid-pregnancy. Masson’s trichrome stained sections show decidual tissue in the uterus of sedentary (**A**,**E**,**C**,**G**) and exercised (**B**,**F**,**D**,**H**) mice. Quantification of vessel lumen area (**I**), lumen diameter (**J**), wall thickness (**K**) and the ratio of lumen area to total vessel area (**L**) for sedentary (S) and exercised (E) CBA/J x BALB/c mice (circles, **A**,**B**,**E**,**F**) or abortion-prone CBA/J x DBA/2J mice (triangles, **C**,**D**,**G**,**H**) in mid-pregnancy (average of 2–5 vessels per dam; *N* = 5–20 dams/group). Data was analysed by linear mixed model including litter size as a covariate. Symbols show outcomes from each dam and bars and whiskers indicate mean ± SD within each group. Differences between male genotypes are indicated by *P < 0.05; **P < 0.01.

Spiral artery lumen area (Fig. 2A–I) and lumen diameter (Fig. 2J) were unaffected by exercise (*P* = 0.059 and *P* = 0.063) or male partner genotype (*P* = 0.220 and *P* = 0.382). The wall thickness (Fig. 2K) and vessel to lumen area ratio (Fig. 2L) of DBA/2J-mated dams were 21% and 35% smaller than BALB/c-mated dams (*P* = 0.024 and *P* = 0.003 respectively) and were unaffected by exercise (*P* = 0.286 and *P* = 0.055 respectively).

### Mid-pregnancy uterine natural killer (uNK) cell abundance

**Fig. 3:**
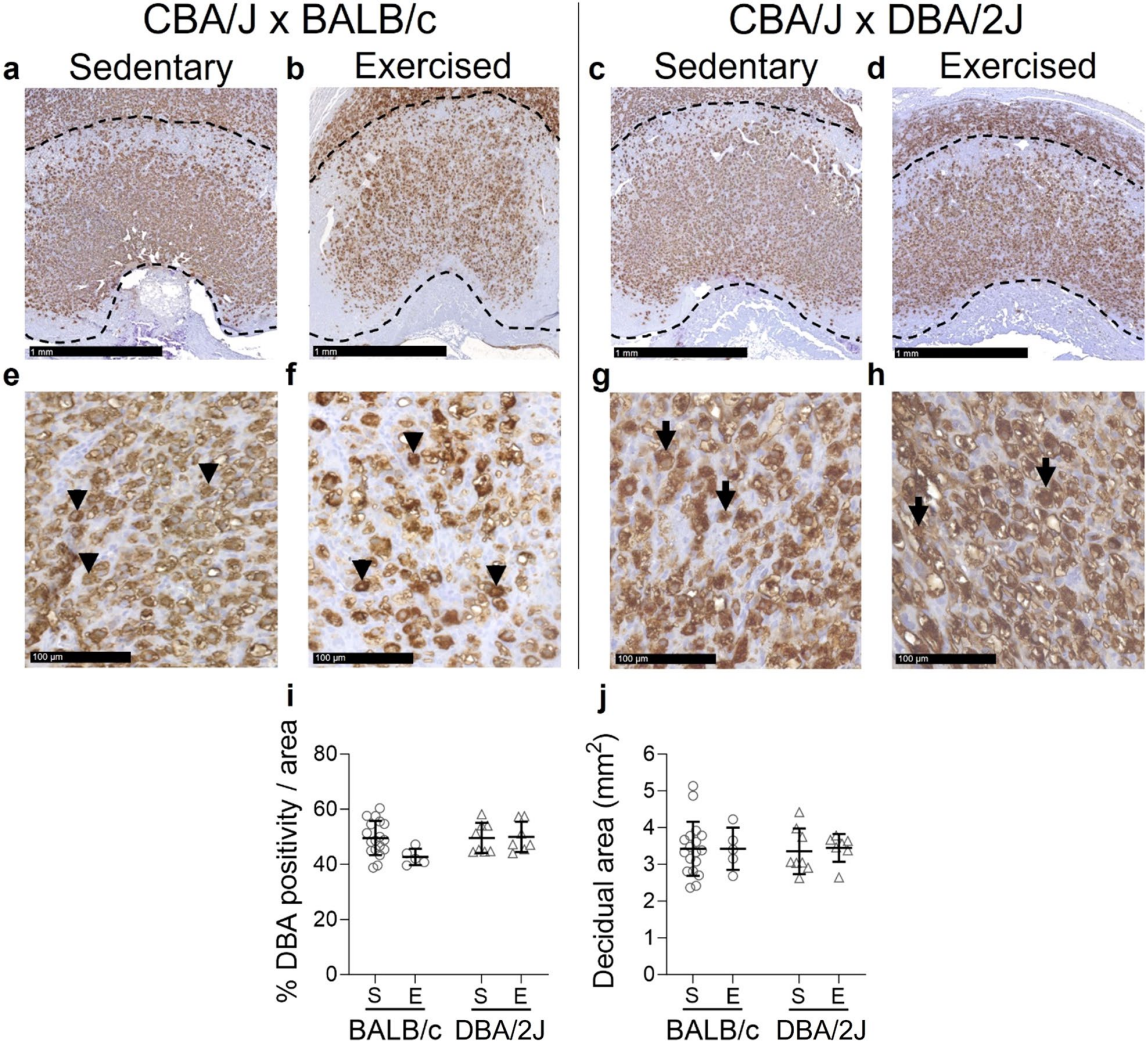
Impact of exercise and paternal genotype on the proportion of uterine natural killer cells in the decidua at mid-pregnancy. DBA lectin-stained sections show decidual areas of sedentary (**A**,**E**,**C**,**G**) and exercised (**B**,**F**,**D**,**H**) mice. Quantification of the DBA^+^ uNK cells are identified (**I**) within the total decidual area (**J**) for sedentary (S) and exercised (E) CBA/J x BALB/c mice (circles, **A**,**B**,**E**,**F**) and abortion-prone CBA/J x DBA/2J mice (triangles, **C**,**D**,**G**,**H**) in mid-pregnancy (*N* = 5–18 dams/group). Data was analysed by linear mixed model including litter size as a covariate. Symbols show outcomes from each dam and bars and whiskers indicate mean ± SD within each group. Differences between male genotypes are indicated by *P < 0.05; **P < 0.01. Arrowheads define DBA^+^ uNK cells.

Uterine NK cells are key components in the pathway by which decidual spiral arteries are remodelled in early pregnancy^7,8^ and their numbers and function are modulated by the maternal immune response to fetal and placental antigens and immune regulators^41^. At GD10.5, the decidual *Dolichos biflorus* agglutinin (DBA) lectin-positive uNK cell abundance (Fig. 3A–I) and the decidual area (Fig. 3J) in dams were unaffected by exercise (*P* = 0.148 and *P* = 0.796 respectively) or male partner genotype (*P* = 0.078 and *P* = 0.968).

### Mid- and late-pregnancy uterine and umbilical artery haemodynamics

**Fig. 4:**
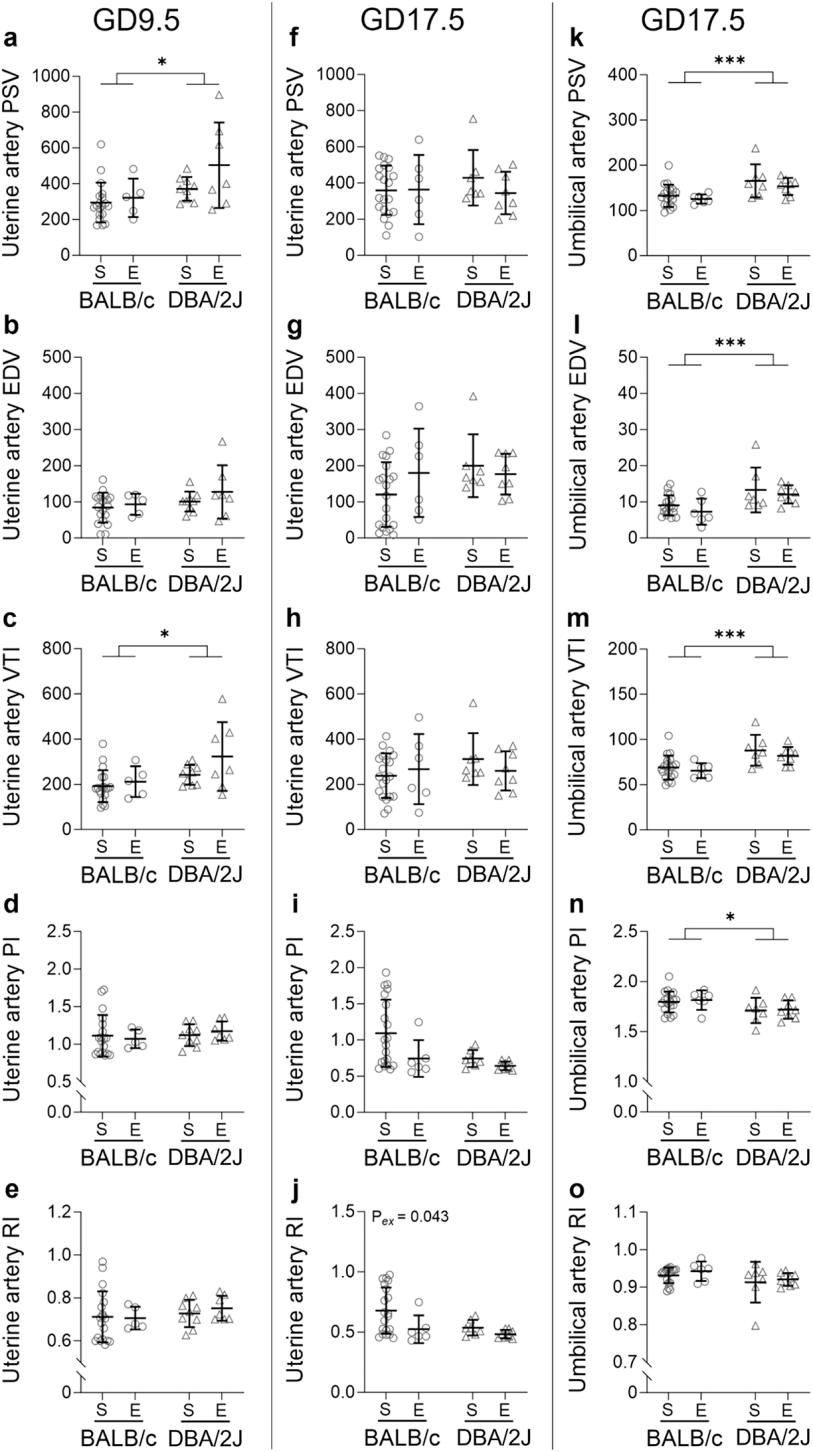
Impact of exercise and paternal genotype on uterine and umbilical artery haemodynamic parameters at mid- and late-pregnancy. Uterine artery hemodynamic parameters at GD9.5 (**A**–**E**) and GD17.5 (**F**–**J**) and umbilical artery hemodynamic parameters at GD17.5 (**K**–**O**) for sedentary (S) and exercised (E) CBA/J x BALB/c mice (circles, **A**,**B**,**E**,**F**) and abortion-prone CBA/J x DBA/2J mice (triangles, **C**,**D**,**G**,**H**) with *N* = 5–20 dams/group. Data was analysed by linear mixed model including litter size as a covariate. Symbols show outcomes from each dam and bars and whiskers indicate mean ± SD within each group. Differences between male genotypes are indicated by *, *P* < 0.05; ***, *P* < 0.001; annotations (P_*ex*_) show significant effects of exercise; PSV; peak systolic velocity, EDV; end diastolic velocity, VTI; velocity time index, PI; pulsatility index, RI; resistance index.

At GD9.5, uterine artery peak systolic velocity (PSV), end diastolic velocity (EDV), velocity time interval (VTI), pulsatility (PI) and resistance index (RI; Fig. 4A–E) were unaffected by exercise (*P* = 0.171, *P* = 0.336, *P* = 0.160, *P* = 0.988 and *P* = 0.798 respectively). Uterine artery EDV, PI and RI were unaffected by male partner genotype (*P* = 0.130, *P* = 0.491 and *P* = 0.372 respectively), but the PSV (Fig. 4A) and VTI (Fig. 4C) were 41% and 21% larger in DBA/2J-mated dams compared to BALB/c-mated dams (*P* = 0.012 and *P* = 0.014 respectively).

At GD17.5, uterine artery PSV, EDV, VTI and PI (Fig. 4F–I) were unaffected by exercise (*P* = 0.411, *P* = 0.534, *P* = 0.759, and *P* = 0.062 respectively) or male partner genotype (*P* = 0.582, *P* = 0.197, *P* = 0.357 and *P* = 0.065 respectively). The uterine artery RI (Fig. 4J) at GD17.5 was 22% smaller in exercised compared to sedentary dams (*P* = 0.043) but was unaffected by male genotype (*P* = 0.074).

At GD17.5, umbilical artery PSV, EDV, VTI, PI and RI (Fig. 4K–O) were unaffected by exercise (*P* = 0.255, *P* = 0.281, *P* = 0.307, *P* = 0.648 and *P* = 0.332 respectively). However, umbilical artery PSV, EDV and VTI (Fig. 4K–M) of DBA/2J-mated dams were 23–56% higher than BALB/c-mated dams (*P* = 0.001, *P* = 0.001 and *P* < 0.001 respectively). Furthermore, the umbilical artery PI (Fig. 4N) was 5% lower in DBA/2J-mated compared to BALB/c-mated dams (*P* = 0.034), although umbilical artery RI was unaffected by male partner genotype (*P* = 0.086).

### Late-gestation pregnancy outcomes

**Fig. 5:**
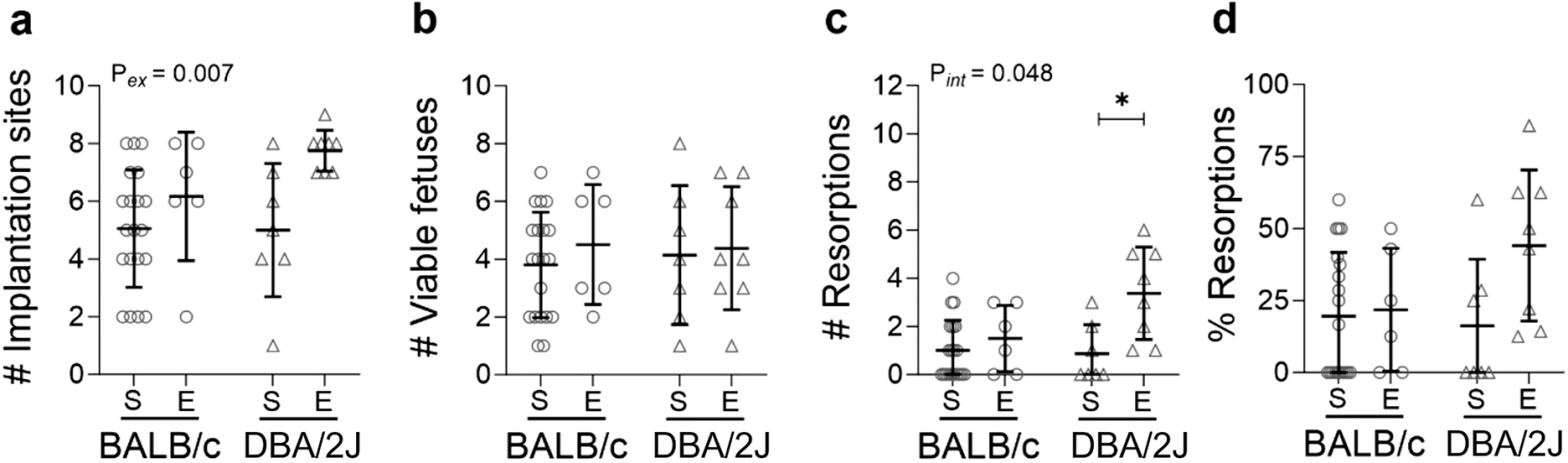
Impact of exercise and paternal genotype on pregnancy outcomes at late-pregnancy. The total number of implantation sites (**A**), number of viable fetuses (**B**), number of resorptions (**C**) and the proportion of resorptions (**D**) per dam for sedentary (S) or exercised (E) CBA/J x BALB/c mice (circles) or abortion-prone CBA/J x DBA/2J mice (triangles) in late pregnancy (*N* = 6–20 dams/group). Data was analysed by linear mixed model. Symbols show outcomes from each dam and bars and whiskers indicate mean ± SD within each group. Annotations (P_ex_) show significant effects of exercise, (P_*int*_) denotes an interaction between male genotype x exercise; *P < 0.05.

Exercise did not affect GD18.5 pregnancy rate measured as a proportion of plugged mice that were pregnant, within either BALB/c-mated dams (sedentary: 21/24 = 88%; exercised: 6/8 = 75%; *P* = 0.399) or DBA/2J-mated dams (sedentary: 7/8 = 87%; exercised: 8/9 = 89%; *P* = 0.929). The total number of implantation sites (Fig. 5A) at GD18.5 was 38% higher in exercised than sedentary dams (*P* = 0.007) and did not differ between male partner genotype (*P* = 0.264). The number of viable fetuses (Fig. 5B) and proportion of resorptions (Fig. 5D) were unaffected by exercise (*P* = 0.512 and *P* = 0.069 respectively) or male partner genotype (*P* = 0.878 and *P* = 0.243 respectively). Effects of exercise on numbers of resorptions (Fig. 5C) at GD18.5 differed according to male partner genotype (interaction: *P* = 0.048). Within DBA/2J-mated dams, resorption numbers were 1.9-fold higher in exercised compared to sedentary dams (*P* = 0.011) but resorption rate was unchanged by exercise within BALB/c-mated dams (*P* = 0.424). Male genotype did not alter the number of resorptions within exercised (*P* = 0.066) or sedentary dams (*P* = 0.802).

### Late-gestation fetal and placental outcomes

**Fig. 6:**
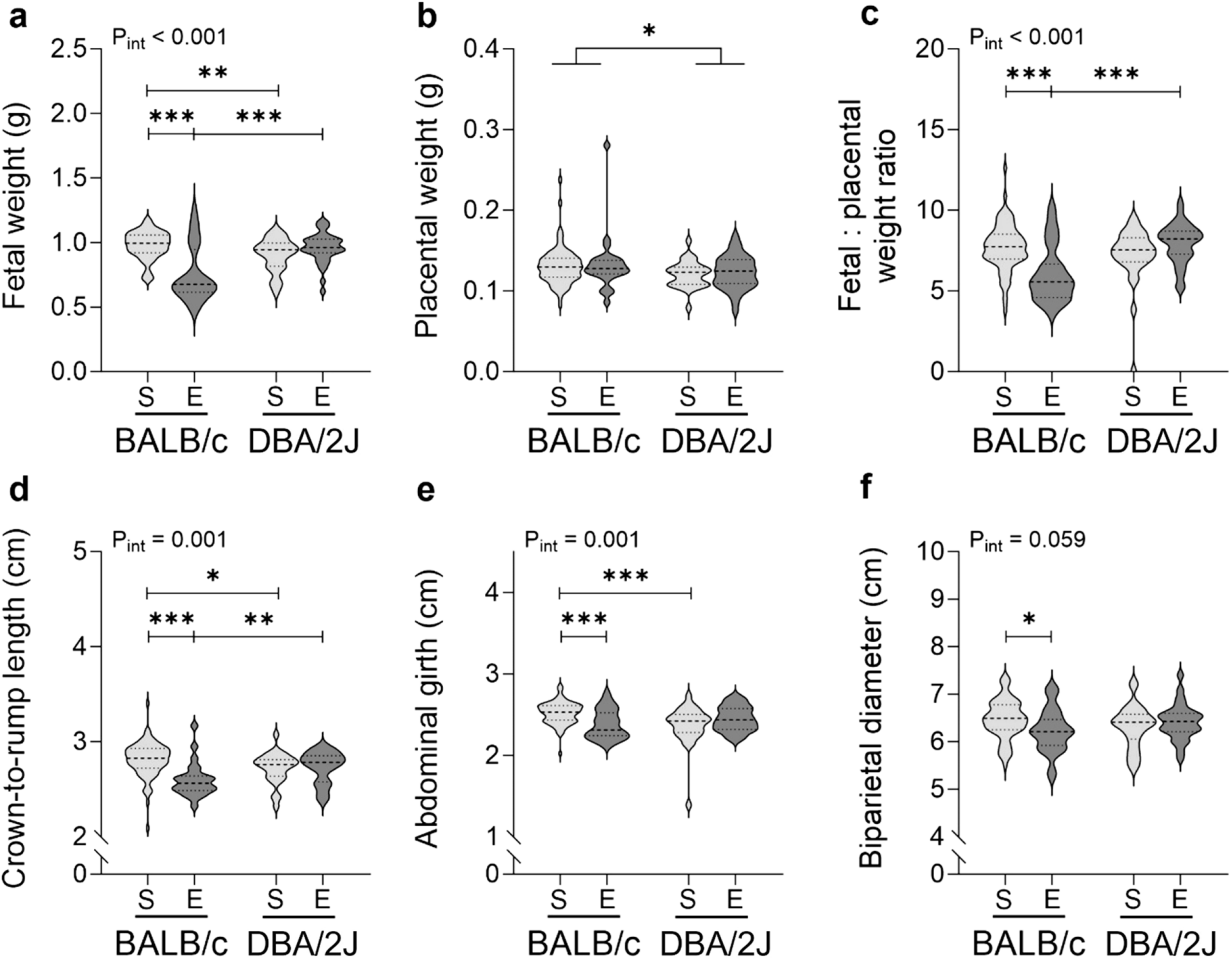
Impact of exercise and paternal genotype on fetal outcomes at late-gestation. Fetal weight (**A)** and placental weight (**B**), fetal: placental weight ratio (**C**), fetal crown-to-rump length (**D**), abdominal girth (**E**), and biparietal diameter (**F**) of concepti were measured in sedentary (S) or exercised (E) CBA/J x BALB/c mice (left) or abortion-prone CBA/J x DBA/2J mice (right) in late pregnancy. *N* = 20 sedentary (1–7 pups/dam) and *N* = 6 exercised (2–7 pups/dam) CBA/J x BALB/c dams, and *N* = 7 sedentary (1–8 pups/dam) and *N* = 8 exercised (1–7 pups/dam) abortion-prone CBA/J x DBA/2J dams. Data were analysed by linear mixed model with dam as subject and litter size as a covariate, and data from individual litter mates were treated as repeated measures on the dam. Data shown as violin plots with mean ± SD. P_*int*_ denotes an interaction between male genotype x exercise; *P < 0.05, **P < 0.01, ***P < 0.001.

At GD18.5, placentas (Fig. 6B) were 9% heavier in DBA/2J-mated dams than in BALB/c-mated dams (*P* = 0.027), and unaffected by exercise (*P* = 0.493). Effects of exercise on fetal weight (Fig. 6A), fetal: placental weight ratio, crown-to-rump length, abdominal girth and biparietal diameter (Fig. 6C–F) at GD18.5 differed according to male partner genotype (interaction: *P* < 0.001, *P* < 0.001, *P* = 0.001, *P* = 0.001 and *P* = 0.059 respectively). Within BALB/c-mated dams, fetal weights, fetal: placental weight ratios, crown-to-rump lengths, abdominal girths and biparietal diameters (Fig. 6A,C–F) were 24%, 22%, 7%, 6% and 4% smaller respectively in exercised compared to sedentary dams (*P* < 0.001 for others, *P* = 0.014 for biparietal diameter). These measures of fetal size were unaffected by exercise in DBA/2J-mated dams (*P* = 0.085, *P* = 0.053, *P* = 0.682, *P* = 0.150 and *P* = 0.379 respectively). Within sedentary groups, fetal weights, crown-to-rump lengths, and abdominal girths were 3–8% smaller in fetuses from DBA/2J-mated compared to BALB/c-mated dams (*P* = 0.002, *P* = 0.020 and *P* < 0.001 respectively) but fetal: placental weight ratios and biparietal diameters were unaffected by male partner genotype (*P* = 0.137 and *P* = 0.104). Within exercised groups, fetal weights, fetal: placental weight ratios and crown-to-rump lengths were 28%, 31% and 5% larger in fetuses from DBA/2J-mated dams compared to BALB/c-mated dams (*P* < 0.001, *P* < 0.001 and *P* = 0.007 respectively) but abdominal girths and biparietal diameters were unaffected by male genotype (*P* = 0.143 and *P* = 0.107).

### Late-gestation placental structure

**Fig. 7:**
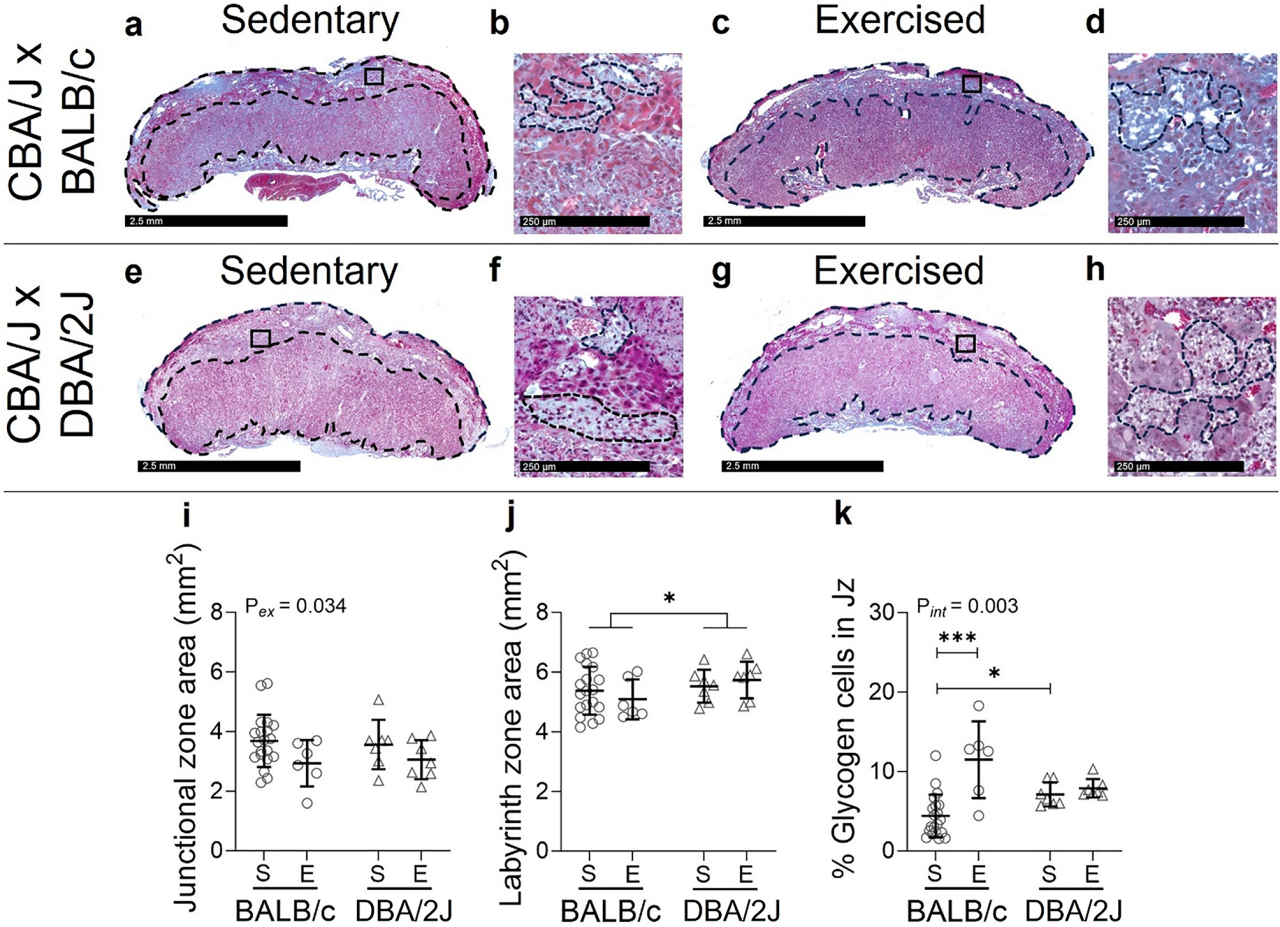
Impact of exercise and paternal genotype on placental structure and the proportion of glycogen cells in the junctional zone of the placenta at late-gestation. Masson’s trichrome-stained sections show placental areas of sedentary (**A**,**B**,**E**,**F**) and exercised (**C**,**D**,**G**,**H**) mice. The junctional zone (**I**) and labyrinth zone (**J**) areas and the proportion of clustered glycogen cells within the total junctional zone area (**K**) were quantified for sedentary (S) and exercised (E) CBA/J x BALB/c mice (circles, **A**–**D**) and abortion-prone CBA/J x DBA/2J mice (triangles, **E**–**H**) in late pregnancy (average of 2 placentas per dam; *N* = 6–20 dams/group). Data was analysed by linear mixed model including litter size as a covariate. Symbols show outcomes from each dam and bars and whiskers indicate mean ± SD within each group. Differences between male genotypes are indicated by *P < 0.05; annotations (P_ex_) show significant effects of exercise, (P_*int*_) denotes an interaction between male genotype x exercise; ***P < 0.001.

The junctional zone area (Fig. 7A,C,E,G,I) at GD18.5 was 18% smaller in exercised than sedentary dams overall (*P* = 0.034) and did not differ according to male partner genotype (*P* = 0.999). The labyrinth zone area (Fig. 7J) was 9% larger in DBA/2J-mated dams than in BALB/c-mated dams (*P* = 0.026) and was unaffected by exercise (*P* = 0.948). Effects of exercise on the proportion of glycogen cells within the junctional zone area (Fig. 7B,D,F,H,K) at GD18.5 differed according to male genotype (interaction: *P* = 0.003). Within BALB/c-mated dams, the proportion of glycogen cells was increased 2.6-fold in exercised compared to sedentary dams (*P* < 0.001; Fig. 7A–D,K), whereas the proportion of glycogen cells was unaffected by exercise in DBA/2J-mated dams (*P* = 0.312; Fig. 7E–H,K). Within sedentary groups, DBA/2J-mated dams had a 1.6-fold more glycogen cells than BALB/c-mated dams (*P* = 0.021; Fig. 7A,B,E,F,K), but glycogen cell numbers did not differ between male partner genotypes within exercised groups (*P* = 0.080; Fig. 7C,D,G,H,K). Although exercise reduced placental junctional zone areas and fetal growth in BALB/c-mated dams, there was no significant correlation between these outcomes (*R* = -0.173, *P* = 0.284). Labyrinth zone area was larger in DBA/2J-mated dams whereas the umbilical artery pulsatility index was lower in DBA/2J-mated dams and a moderate negative correlation was identified between these outcomes (*R* = -0.337, *P* = 0.036).

## Discussion

In the present study, an exercise protocol beginning before conception and sustained throughout pregnancy had different impacts on pregnancy outcomes depending on the genotype of the sire and fetus. In an uncomplicated pregnancy generated by mating CBA/J dams with BALB/c sires, exercise caused symmetric fetal growth restriction and increased glycogen cell content in the junctional zone of placentas. In contrast, in abortion-prone pregnancies generated by mating CBA/J dams with DBA/2J sires, exercise increased placental efficiency but also increased numbers of resorptions in late pregnancy. This study confirmed the abortion-prone phenotype of the CBA/J x DBA/2J mating with increased resorptions and inadequate spiral artery remodelling, associated with larger placentas than BALB/c-mated dams. Regardless of sire genotype, exercise increased implantation sites at mid and late pregnancy, reduced body fat ratios and decreased uterine artery resistance. In addition, maternal exercise before and throughout pregnancy decreased the junctional zone areas of placentas, regardless of genotype.

The likelihood of mating progressing to pregnancy and the interval between placing females with males and finding a mating plug was not changed by exercise, regardless of paternal genotype. However, the number of implantation sites at both mid and late pregnancy was increased by exercise regardless of male partner genotype. There is evidence that maternal exercise before and during pregnancy enhances blastocyst formation and in particular, embryonic myogenesis^42,43^, so improved blastocyst and embryonic development may have contributed to greater implantation success with exercise. Our findings in mice share some similarities to human observations, but not complete coherence. In women undergoing IVF treatment, a greater time exercising prior to treatment is associated with retrieval of an increasing number of oocytes, however exercise did not improve in implantation, pregnancy or live birth rates^44^. In a meta-analysis, although maternal exercise before IVF or ICSI cycles did not change implantation rates, it was associated with an increased rate of clinical pregnancy and live birth^45^. While exercise was able to influence implantation rates in mid-pregnancy our study also showed that it altered placental architecture in late pregnancy, resulting in a reduced junctional zone area. Running exercise during pregnancy in women is reported to increase villous vascular volume with speculations of enhanced placental transfer of oxygen and feto-placental growth^28^ and furthermore exercise in human pregnancy did not negatively impact placental weight at term^46^. Our data indicates that exercise leads to reductions in the placental junctional zone area as well as FGR in uncomplicated pregnancies. The junctional zone of the placenta is essential for glycogen storage, which plays a critical role in nutrient and energy transfer to the growing fetus^47^. However, junctional zone area and fetal weight were not correlated in the present cohort. Although exercise reduced placental junctional zone area, there was no impact on overall placental weight. Since the junctional zone is the principal site of hormone production in the mouse, changes to glycogen cell content may nevertheless cause altered placental endocrine function and the modulation of maternal blood flow^48,49^. There is some evidence suggesting exercise before or during pregnancy in women can increase resistance to uteroplacental blood flow^50,51^, but in our study exercise did not reduce uterine artery resistance in late pregnancy in mice. While this finding in women suggests a potential for adverse effects of exercise on placental function, we found uteroplacental blood flow to be overall better with exercise. These findings suggest that exercise can have a positive impact on the implantation rate and uterine artery function despite losses to placental area.

While exercise highlighted benefits to uterine artery blood flow and placental structure, we wanted to investigate the significance of paternal genetic background more closely. Regardless of exercise, we found that DBA/2J-mated dams have greater resorptions in mid-pregnancy and impaired decidual spiral artery remodelling compared to BALB/c-mated dams, consistent with a previous report^40^. However, unlike another study that found reductions in the absolute volume of the placenta and the labyrinth zone but not the junctional zone, we observed larger placentas, specifically a larger labyrinth zone area, in DBA/2J-mated dams compared to BALB/c-mated dams^52^. In addition, DBA/2J-mated dams exhibited lower umbilical artery pulsatility in late pregnancy suggesting a reduced resistance to blood flow and no indication of fetal growth restriction^51^. A moderate negative correlation was identified between labyrinth zone area and the umbilical artery pulsatility index. Since the labyrinth zone is the primary site of maternal-fetal exchange^47^, a larger surface area may allow more efficient blood flow distribution, and lower placental resistance to blood flow, thereby improving the efficiency of nutrient transfer to the fetus. Nevertheless, absence of fetal growth restriction does not exclude the possibility that fetal growth is compromised by early uteroplacental maladaptation. Distinct impairments to decidual spiral artery remodelling at mid-pregnancy within DBA/2J-mated dams, evidenced in the present study by reduced wall thicknesses and vessel to lumen area ratios, is usually associated with a reduced abundance of DBA^+^ uNK cells within the decidua at mid-pregnancy. DBA^+^ and DBA^-^ uNK cells are specific subsets of activated uNK cells essential for spiral artery remodelling that increase in the decidua at mid-pregnancy. While both types are involved in spiral artery remodelling, DBA^+^ uNK cells primarily release angiogenic factors while the DBA^-^ uNK cells express more IFNγ^53^. Surprisingly, our DBA/2J-mated dams showed no difference in their DBA^+^ uNK cell abundance in the decidua of implantation sites compared to BALB/c-mated dams. Nevertheless, these findings indicate that sire genotype is an important determinant of uterine vascular adaptation to pregnancy and fetal outcomes. Despite having the same MHC haplotype, there are other genetic differences between DBA/2J and BALB/c male mice that likely affect fetal and placental growth both directly, and indirectly via the dam’s immune response to pregnancy. An immune-mediated effect is associated with a differing seminal fluid composition in DBA/2J males compared to BALB/c males, which invokes an impaired immune adaptation to pregnancy^37^. Seminal fluid composition as well as the sperm epigenome may contribute to between-male genetic variability that can be observed in humans and mice^54^. Similar mechanisms may underlie the role of paternal genetic risk factors in the development of preeclampsia in women^55^.

In addition to exploring the impact of male genotype overall, we also observed differences in the response to exercise in pregnant CBA/J dams depending on the genotype of the sire from their pregnancies. In uncomplicated pregnancies resulting from BALB/c matings, exercise caused symmetric fetal growth restriction evidenced by proportional reductions in crown-to-rump lengths, abdominal girths and biparietal diameters^56,57^. In some studies maternal exercise in healthy human pregnancy has been associated with decreased fetal weight^58,59^, while others show no change in incidence of low birth weight, but a reduced likelihood of high birth weight after exercise^19^. When relevant studies were considered collectively in a large systematic review it was concluded that maternal exercise was not associated with low birth weight^60,61^, but that the intensity of maternal exercise was negatively associated with birth weight^61^. The difference in response to exercise in healthy human and healthy mouse pregnancies might reflect the greater relative energy requirement of mouse compared to human pregnancy. Maternal weight gain during pregnancy is 20–30% in humans and > 40% in mice^62–64^, with much lower total fetal weight relative to maternal weight in humans than in mice (6% vs. 22%)^62,65^. Furthermore, within these uncomplicated mouse pregnancies, exercise reduced placental function paired with an increased glycogen cell content in the junctional zone. The latter is thought to mark a failure of migration of glycogen cells from the junctional zone into spiral arteries and is associated with fetal growth restriction^66,67^. Conversely, there is evidence indicating low glycogen cell content in the junctional zone of the mouse placenta can be associated with fetal growth restriction^48,68^. Greater placental glycogen cell content as well as trends towards narrower spiral arteries (*P* = 0.06) suggest exercise may impair the uteroplacental vasculature adaptation. Impaired spiral artery remodelling can constrain the supply of oxygen and nutrients to the placenta thereby affecting fetal growth^9^. This may explain why fetal growth is reduced by maternal exercise in uncomplicated pregnancies, but additional studies with a larger sample size are required to confirm this. Our observation of reduced placental function (indicated by a lower fetal: placental weight ratio) in exercised dams is in agreement with a prior study in obese exercised mice, whereby in controls, exercise alone reduced the fetal: placental weight ratio, also causing reduced nutrient supply across the placenta manifesting as reduced fetal blood glucose^33^. The degree of adverse effect of exercise in the uncomplicated pregnancy group was somewhat unexpected, given the consistent reporting of safety of pre-natal and late-pregnancy exercise in uncomplicated human pregnancy^22^ even at vigorous intensities^27^ with respect to fetal outcomes.

This study highlights that the impact of exercise on pregnancy is not uniform and can be significantly influenced by genetic factors. Consistent with previous reports, fetuses carried by sedentary dams were growth-restricted in abortion-prone pregnancies arising from DBA/2J matings, compared to those of uncomplicated pregnancies^38^. In contrast, within exercised dams, fetuses from abortion-prone CBA/J x DBA/2J matings were larger and had greater placental efficiency compared to those from uncomplicated CBA/J x BALB/c matings. This suggests that exercise may confer a benefit to the compromised pregnancies, consistent with exercise-induced improvements in placental vascular density and metabolic outcomes reported in other mouse models of complicated pregnancies^33,69^. The mechanistic basis for the differential effects of exercise that we observed in DBA/2J-mated mouse pregnancies with impaired immune function is not clear, as exercise modulates many components of the immune system and can have both pro- and anti-inflammatory effects. The process of exercise activates the release of free calcium and the synthesis of pro-inflammatory cytokines such as tumour necrosis factor (TNF) and interleukin-1 beta (IL-1β) which can then regulate endothelial cells to attract neutrophils to the muscle fibres. Minor tissue damage caused by exercise causes elevated synthesis of IL-6 and IL-8 cytokines that stimulate the activation and release of reactive oxygen species. Running exercise in particular leads to the greatest increase in IL-6^70^. Exercise directly promotes the generation of Treg cells, acting to promote thymic Treg accumulation with consequences for the kinetics of muscle repair and growth^71^. This can shift the immune balance towards a reduced Th1/Treg profile. Acute exercise can cause a temporary depression of immune function for 3–24 h after depending on the exercise intensity and duration^70^. Therefore, the stress of exercise in the abortion-prone model could shift the Th1/Treg balance and confer a benefit to the outcome of pregnancy. Whether similar effects might be seen in humans with complicated pregnancies is unclear as ethical constraints prevent intervening to evaluate exercise in these pregnancies. It seems possible that interactions between exercise and the altered pregnancy immune response could contribute to the different effects seen in the CBA/J x BALB/c mating combination, compared to the CBA/J x DBA/2J mating combination.

The exercise protocol used in this study was selected following a pilot study and based on published studies in pregnant and non-pregnant female mice^10,33,34,72^. Exercise intensity was slowly increased over 4 weeks to a final duration of 30 min, 5 days a week, which was maintained for the 2 weeks prior to pregnancy (6 weeks of pre-mating exercise in total) and continued throughout pregnancy. This protocol is considered moderate exercise intensity, based on a previous report that non-obese pregnant mice undergoing treadmill exercise at similar speeds (10–14 m/min) achieved maximal oxygen consumption rates (VO2) of 40–65% depending on the stage of pregnancy^33^. At the end of the protocol, relative fat masses and fat to muscle ratios in late pregnancy were substantially lower in exercised than sedentary dams regardless of genotype, demonstrating systemic effects of the intervention. We maintained the same exercise protocol in the pregnant mice as was used in studies on obese pregnant mice^34,73^, although our study had a longer pre-pregnancy acclimatisation period. Other studies have reduced exercise duration and intensity towards the end of pregnancy to accommodate maternal physical changes^33,73^. Similarly, the duration of voluntary wheel running exercise in rodents naturally tapers off as pregnancy progresses^74,75^. Our protocol can therefore be considered moderate exercise, consistent with recommendations for exercise during human pregnancy^76^. We consider the use of treadmill running to be a strength of the study, since it enabled consistent intensity, and accurate measurement of distances and duration of exercise for each mouse. However, regular running is a very specific form of cardiovascular exercise and the potential for different effects of other types of aerobic and resistance exercise needs to be considered. There are several limitations in the utility of mice as a model for exercise in human pregnancy. The response to exercise of varying intensity and duration obviously varies greatly between the species. We recognise that the controlled exercise regime we utilized may not accurately replicate the human experience of exercise in pregnancy, particularly considering that humans exhibit a wide range of fitness levels and exercise preferences. Metabolic rate in mice is much less than in humans due to their smaller body mass – giving rise to different energy requirements during pregnancy^77^. Furthermore, the short pregnancy duration of 19–21 days in mice allows a substantially shorter time for adaptation in comparison to the 40-week gestation period in women, allowing a more prolonged and complex adaptative process in human pregnancy^47^.

In summary, this study was the first to demonstrate that exercise in uncomplicated and abortion-prone mouse pregnancies can be impacted by paternal genetics. Our findings show that in abortion-prone pregnancies, exercise led to increased fetal resorptions, no change in the number of viable fetuses, and surviving fetuses were not growth restricted. Whereas in uncomplicated pregnancies, exercised dams had symmetrically growth restricted fetuses possibly a result of altered placental development. Overall exercise mostly had a positive impact with consistently increased implantations, reduced uterine artery resistance and body fat reductions, with a reduction in junctional zone area that appeared not to detrimentally affect overall pregnancy outcome. As expected, regardless of exercise, DBA/2J-mated dams had a greater number of resorptions, poor spiral artery remodelling, and larger placentas compared to BALB/c-mated dams. Nevertheless, this study indicates exercise more substantially affected the fetal outcomes of the uncomplicated pregnancies than the abortion-prone, indicating paternal genetics impact physiological state in pregnancy. Other mouse studies of exercise in pregnancy do not specify the male genotype, and have not considered its impact^34,35,42,73,75^. In many of these studies it seems likely that the males were of the same genotype as the females, a state that is not relevant to human pregnancies and so further emphasises the need to consider sire genetics in future studies. The impacts of exercise appear to interact with paternally derived semen and fetal factors, that may be mediated via the maternal immune response. There is thus potential for both maternal, paternal, and fetal factors to affect the impacts of exercise on pregnancy outcome. Future research is needed to assess the variation in type, intensity and/or timing of exercise, and to tease apart the interactions between exercise, fetal sex, and pregnancy health in litter-bearing species, and their underlying mechanisms.

## Methods

### Animals and treatments

Virgin female CBA/J mice were bred at an established colony in the University of Adelaide specific pathogen-free facility (using founder CBA/J mice purchased from Jackson Laboratory, Bar Harbor, ME, Unites States). All experimental mice were housed in the University of Adelaide conventional facility. Male BALB/cArc (BALB/c) and DBA/2JArc (DBA/2J) mice were purchased from Animal Resources Centre (Perth, Western Australia). All mice were housed at 40–60% humidity, 12 h:12 h light: dark cycle, 21–22 °C, and had *ad libitum* access to standard rodent chow (Irradiated 2920X, Envigo) and water. After at least 1 week of acclimatisation in the conventional facility, cages of 3–5 females aged 6–8-weeks (initial weight 15–19 g), were alternately allocated to remain sedentary or undergo exercise on the conveyor belt of a motor-driven treadmill (Panlab, Barcelona, Spain) from 6 weeks prior to mating and throughout gestation. Exercise durations and intensities (Table 2) were based on those previously published in female pregnant and non-pregnant mice^10,33,34,72^. Females were progressively familiarised to running for 4 weeks until they reached a maximum intensity (30 min at 10 m/min, 5 days per week, 0% incline) that was then maintained for 2 weeks prior to mating (Table 2). Exercise was completed between 0900 and 1400 h on experimental days, with 5-minute warm up and cool down periods at 5 m/min at the beginning and end of each session. An air puff was activated to encourage stationary and less compliant mice to exercise. Mice were checked daily and clinical record sheets were kept, as per our welfare requirements. Mice were rested if they did not attempt to run for the first half of the protocol on that day. Sedentary control mice were placed on a stationery treadmill with supervision for the same total durations as exercised mice. On the day following completion of the 6-week pre-mating exercise protocol, females were alternately allocated to housing with a proven-fertile BALB/c or DBA/2J stud male (1 male: 1 female per cage). Mating was confirmed by detection of a vaginal plug in the morning, which was defined as gestational day (GD) 0.5. Mated females were removed from males and pair-housed for the remainder of the study. Female mice not mated within 96 h of being housed with a male were excluded from the study. Pregnant mice ceased exercise 2 days before being humanely killed by cervical dislocation, to avoid potential confounding by acute effects of exercise. A subset of female mice mated to BALB/c males (*N* = 5 exercised, *N* = 10 sedentary) and DBA/2J males (*N* = 7 exercised, *N* = 9 sedentary) were allocated to be humanely killed by cervical dislocation on GD10.5. A separate subset of female mice mated to BALB/c males (*N* = 6 exercised, *N* = 8 sedentary) and DBA/2J males (*N* = 7 exercised, *N* = 8 sedentary) were allocated to be humanely killed by cervical dislocation on GD18.5. Due to low sample size, an additional cohort of sedentary BALB/c-mated mice, studied immediately after the first cohort, were added to the present study (*N* = 10 to GD10.5 and *N* = 12 to GD18.5). Mice that were mated but were not pregnant at the time of endpoint experiments were excluded from the study (*N* = 20/87). Mice were excluded from the final analysis if they had less than 1 viable implantation site. Compliant mice ran a total distance of 6900–8900 m for mice humanely killed at GD10.5 and a total distance of 7300–8900 m for mice humanely killed at GD18.5. Exercised mice that ran less than 6000 m in total throughout the 6 weeks were considered non-compliant with the exercise intervention and therefore excluded from analysis (*N* = 1/28).

**Table 2:** Exercise protocol before and during pregnancy.

	Week		Duration (min)	Speed (m/min)
Pre-pregnancy	1	Mon/Wed/Fri	10	10
		Tues/Thurs	0	0
	2	Mon	10	10
		Tue-Fri	15	10
	3	Mon	15	10
		Tue-Fri	20	10
	4	Mon	20	10
		Tue-Thurs	25	10
		Fri	30	10
	5–6	Mon-Fri	30	10
During pregnancy	GD0.5-1.5	Mon-Fri	0	0
During pregnancy	GD1.5-15.5	Mon-Fri	30	10

### Pregnancy outcomes, fetal sex determination and tissue collection on GD10.5 and 18.5

At GD10.5, the uterus was visually inspected to determine the number of normal and abnormal implantation sites. Abnormal implantation sites were defined as visibly smaller and more anaemic than others in the litter^80^. To maintain artery structure, the uterine artery was tied off with dental floss distal to each ovary and adjacent to the cervix^81^. The uterus containing implantation sites was dissected and fixed in 4% paraformaldehyde, processed, and embedded in paraffin wax. At GD18.5, the number of fetuses and resorptions^80^ were counted, and fetuses and placentas were dissected and weighed. Resorptions were visualised as small black masses in between growing fetuses^80^. Fetal crown-to-rump length, abdominal girth, and biparietal diameter were measured. Two representative placentas from healthy fetuses were collected, bisected, fixed in 4% paraformaldehyde, processed, and embedded in paraffin wax for subsequent analysis. Fetal tails were snap-frozen, and fetal sex was later determined by PCR for the Y chromosome specific gene SRY (forward primer 5’ AACAACTGGGCTTTGCACATTG 3’, reverse primer 5’ GTTTATCAGGGTTTCTCTCTAGC 3’), utilising Myogenin (forward primer 5’ TTACGTCCATCGTGGACAGC 3’, reverse primer 5’ TGGGCTGGGTGTTAGTCTTA 3’) as a control gene^82^. To assess effects of exercise on body composition, maternal left and right gastrocnemius, quadriceps, biceps, and triceps muscles were collected and weighed, and maternal retroperitoneal fat pads were weighed.

### Histological analysis of implantation sites and placentas

Histological analyses were performed blinded to maternal exercise and genotype. Two adjacent normal implantation sites (GD10.5) per dam were serially sectioned (7 μm), and stained with Masson’s trichrome for analysis of implantation site decidual spiral artery morphology^81^. Slides were imaged at 40x magnification using a Nanozoomer-XR Digital Slide Scanner (Hamamatsu Photonics). Three sections at 49 μm apart were assessed in 2–5 vessels per implantation site from the middle two quadrants of each implantation site. For each section, the total vessel area, lumen area and lumen circumference were measured to calculate vessel diameter and wall thickness. To visualise uNK cells within GD10.5 implantation sites, tissue sections were stained using *Dolichos Biflorus* Agglutinin (DBA) lectin (Vector Laboratories), counterstained with Weigert’s haematoxylin^83^. The DBA-positive area as a proportion of decidual area was measured using Fiji ImageJ version 2.0.0. in 2 sections per implantation site and 2 implantation sites per dam.

Placentas from GD18.5 pregnancies were bisected at the mid-sagittal plane, then sectioned (5 μm) and stained with Masson’s Trichrome following standard protocols^84^. Areas of placental junctional zone and labyrinth zone were measured, and total cross-sectional area and the proportion of junctional zone to labyrinth zone calculated for each placenta. Glycogen cells within the junctional zone were identified on the basis of their large size and characteristic pale blue staining, and quantified as a proportion of total junctional zone area^82^.

### Ultrasound biomicroscopy

Mice were anesthetised with 3% isoflurane in oxygen on GD9.5 or GD17.5 and ultrasound biomicroscopy of the uterine artery was performed^82,85^. On GD17.5, umbilical artery waveforms from at least three different fetuses per litter were also assessed^85^. Mice were returned to cages placed on a warming pad and monitored continuously until mobile, then returned to the conventional facility. Average peak systolic velocity (PSV), end diastolic velocity (EDV) and velocity time index (VTI) were measured from three cardiac cycles per dam or fetus. Resistance index (RI=(PSV-EDV)/PSV) and pulsatility index (PI=(PSV-EDV)/VTI) were calculated^85^.

### Statistical analysis

Data were analysed with IBM SPSS Statistics software for Windows (version 25). The proportion of plugged mice that were pregnant at autopsy was compared between exercised and sedentary groups by Chi square tests. Effects of exercise and paternal genotype on numbers of total and viable implantation sites and resorptions (GD18.5) or abnormal implantation sites (GD10.5) were assessed by mixed model analysis. Effects of exercise and paternal genotype on measures of maternal body composition, vascular remodelling, haemodynamic parameters, and placental histology were assessed by mixed model analysis, including litter size as a covariate. Effects of exercise and paternal genotype on fetal and placental size were assessed by mixed model analysis, treating the data for each conceptus as a repeated measure of the dam and including litter size as a covariate. To test the a priori hypothesis that effects of exercise differ between healthy and compromised pregnancies, sub-analyses were performed where interactions between male genotype and exercise were evident at *P* < 0.1. Relationships between placental labyrinth zone area and umbilical artery pulsatility index and between placental junctional zone area and fetal weight were assessed by Pearson’s correlation. Data are presented as mean ± SD. *P* < 0.05 was considered statistically significant, and N values reflect the number of dams.

## Data Availability

All analysed data that support the findings of this study are present in the manuscript, raw data sets are available upon reasonable request from the corresponding author.
